# Editorial Department

**Published:** 1863-04

**Authors:** 


*Editors of Medical Journals and the so-called Dermatobiotikon:*

So far as this instrument being capable of sticking into the skin about a dozen needles at the same time, by means of their attachment to a spring, and thus of introducing some such articles as Croton Oil and Antimony, we have nothing in particular to say; admitting this, we regard it as practically absolutely worthless, since the occasion for such medication is but very rare, and when indicated is better accomplished by the painless plan of simple application. The inventor of this instrument has also invented a book much more remarkable in its construction, setting forth the merits of this medication and the *modus operandi* of cure for a great variety of diseases. This book requires no letters patent to protect it; it could not be imitated or equaled in absurdity. Editors who receive this instrument may know that in Buffalo this machine is used as a *speciality* when all further comment will be unnecessary.

